# Editorial for the Special Issue on Magnetic and Spin Devices, Volume II

**DOI:** 10.3390/mi14112131

**Published:** 2023-11-20

**Authors:** Viktor Sverdlov, Seung-Bok Choi

**Affiliations:** 1 Christian Doppler Laboratory for Nonvolatile Magnetoresistive Memory and Logic, Institute for Microelectronics, TU Wien, 1040 Vienna, Austria; 2 Department of Mechanical Engineering, The State University of New York at Korea (SUNY Korea), 119 Songdo Moonhwa-Ro, Yeonsu-Gu, Incheon 21985, Republic of Korea; 3 Department of Mechanical Engineering, Industrial University of Ho Chi Minh City, Ho Chi Minh City 7000, Vietnam

Although the miniaturization of metal–oxide–semiconductor field effect transistors (MOSFETs)—the main driver behind an outstanding increase in the speed, performance, density, and complexity of modern integrated circuits—is continuing, numerous outstanding technological challenges in complimentary metal–oxide–semiconductor (CMOS) device miniaturization are slowly bringing the downscaling to saturation. As the CMOS devices employed intrinsically rely on electron charge for their operation, to slow down the saturation of CMOS scaling, researchers are looking to an additional property of electrons: electron spin. Electron spin is attracting increasing attention as a suitable candidate for complementing or even replacing electron charge in future microelectronic devices [1,2,3].

Because of the success of the initial Special Issue on Magnetic and Spin Devices [4], we have decided to continue addressing the ever-advancing progress in spin-driven and magnetic devices with this second volume. There are 10 research articles published in this Special Issue, covering recent advances in research aspects ranging from manufacturing magnetic materials to modeling techniques for highly scaled magnetoresistive devices, design and optimization approaches, complex magnetic systems, and sensing applications.

Electron spin is characterized by two projections on a given axis. The axis is typically defined by a magnetic field or by a magnetization direction when the material is a ferromagnet. The magnetization in a ferromagnet itself can be used to store information when the ferromagnet possesses uniaxial magnetic anisotropy: its magnetization can be aligned along the anisotropy axis in either direction. In *Contribution 1*, a simple method to grow thin ferromagnetic films of permalloy (Py), an alloy of 80% Ni and 20% Fe, on a silicon wafer via electrochemical deposition [5] is explored. The effect of Py thickness on the magnetic properties of thin films was investigated by using field emission scanning electron microscopy, energy-dispersive X-ray spectroscopy, atomic force microscopy, ferromagnetic resonance, anisotropic magnetoresistance, and the magneto-optic Kerr effect. The magnetoresistance ratios of the deposited Py thin films were around 0.23%, while the damping constant was 1.36 × 10^−2^; these characteristics are comparable to those of expensive sputtered layers.

At all stages of semiconductor device development, accurate technology computer-aided design (TCAD) tools are paramount to predict the device functionalities, to optimize the parameters, and to obtain the best performance. In particular, process and device TCAD has become indispensable in the design cycle of novel devices and technologies [6]. A comprehensive simulation approach capable of accurately describing the complex structures and behaviors of emerging ultra-scaled magnetoresistive random access memory (MRAM) devices [7] is proposed in *Contribution 2*. The approach is based on the finite element method solution of the Landau–Lifshitz–Gilbert equation coupled to the spin and charge transport through the device, and it allows for predicting the switching behavior of recently proposed structures with a double reference layer [8] and MRAM cells with an elongated and composite free layer [9]. The modeling approach was extended in *Contribution 3* to investigate the role of temperature on the switching behavior in a spin-orbit torque (SOT) MRAM cell. It was demonstrated that the critical voltage for SOT switching is significantly reduced at elevated temperatures. The critical SOT voltage displayed a parabolic decrease as a function of the voltage applied across the magnetic tunnel junction (MTJ) of the SOT-MRAM cell, in agreement with recent experimental data [10].

Magnetic tunnel junctions switch stochastically [11], with their switching probabilities dependent on the applied current. *Contribution 4* proposes the use of electrically controlled MTJs to mimic Ising spins to build an Ising annealing machine [12]. Simulations with an Ising annealing system constructed using 64 such spins demonstrated factorization of *n*-bit integers up to 2^64^ with a temporal complexity proportional to $\sqrt{n}$.

Heat-assisted magnetic recording (HAMR) technology is a promising alternative to replace the current perpendicular magnetic recording, further increasing the areal density of hard disk drives; however, unwanted noise may affect the read-back signal. The transition jitter noise at ultrahigh areal density in *L*1_0_-FePt-based [13] HAMR technology was explored by means of micromagnetic simulations in *Contribution 5*. The lowest transition jitter was obtained in an 8 nm track width at a 9 nm bit length, yielding a projected ultrahigh areal density of 8.9 Tb/in^2^ for upcoming applications.

Regarding the design and optimization of a complex electromagnetic element, namely, a solenoid valve used to control the flow velocity and the flow direction in microfluidics [14], *Contribution 6* deals with a numerical determination of the magnetic isolation ring position in a solenoid valve for optimal dynamic response performance. The proposed model was verified experimentally, and optimally designed solenoid valves were realized.

In *Contribution 7*, a novel method for antenna miniaturization based on acoustic excitations and magnetoelectric coupling [15] is elaborated. A magnetoelectric antenna was designed and successfully fabricated and tested. The results demonstrate its potential to overcome the miniaturization limits and impedance mismatch of traditional antennas.

Composite multiferroic materials comprising piezoelectric and magnetostrictive properties [16] are also suitable for designing novel types of interconnects for efficient magnetic signal transmission. Internal magnetic signal amplification due to a portion of energy being transferred from electric to magnetic domains via stress-mediated coupling helps to efficiently compensate strong signal dumping so that the amplitude of the magnetic signal remains constant during the propagation. The model introduced in *Contribution 8* predicts the group velocity of a magnetic signal up to 10^5^ m/s with an amazingly small energy dissipation of less than 10^−18^ J per bit per 100 nm.

Exploring magnetostrictive materials’ properties has great potential for the development of advanced sensors. Magnetorheological elastomers (MREs) are a representative example of such sensors; these materials’ mechanical properties can be controlled by the use of magnetic fields with enhanced magnetostriction magnitude and reaction force [17]. In *Contribution 9*, an approach based on exploring materials with storage modulus above 300 kPa was pursued by investigating various compositions of carbonyl iron particles. It was demonstrated that increments in both the magnetostriction percentage and normal force were achieved at a higher concentration of the particles. This behavior confirms the feasibility of sensor applications of magnetorheological elastomers.

A specially designed magnetic field gradiometer based on a single elliptical planar Hall effect sensor was fabricated and tested in *Contribution 10*. The gradiometer measured magnetic fields at nine different positions within a length scale of a few millimeters. It was demonstrated that this innovative device is able to detect magnetic field gradients with equivalent gradient magnetic noises of about 958, 192, 51, and 26 pT/(mm√Hz) at 0.1, 1, 10, and 50 Hz frequencies, respectively. The fabricated low-cost, high-resolution, and small-footprint gradiometer has great potential for portable and wearable applications, as well as for body implants [18].

We would like to sincerely thank all the authors for submitting exceptional research papers to this Special Issue. We would also like to thank all the reviewers who took their precious time to carefully examine and help improve the quality of all submitted manuscripts. Last, but not least, we would like to thank Ms. Aria Zeng, the Section Managing Editor, for her outstanding continuous support.
